# Supplementary material for: NEDD9 Overexpression Causes Hyperproliferation of Luminal Cells and Cooperates with HER2 Oncogene in Tumor Initiation: A Novel Prognostic Marker in Breast Cancer
Source: Cancers (Basel). 2023 Feb 9;15(4):1119. doi: 10.3390/cancers15041119 (PMC9954084; doi:10.3390/cancers15041119)
Supplement: Supplementary file 1 [file cancers-15-01119-s001.zip › cancers-2150513-supplementary.pdf]

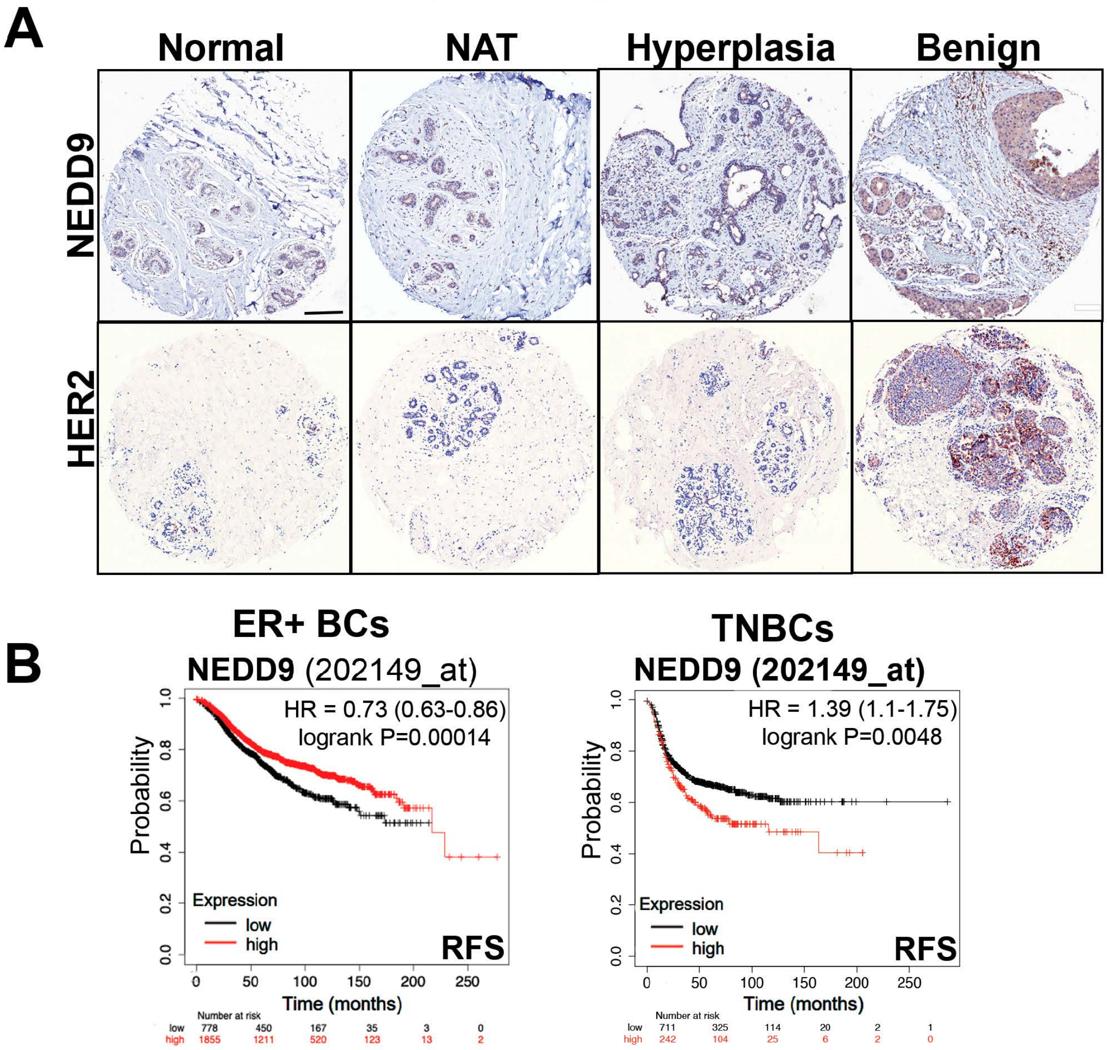

Figure S1: A Immunohistochemistry Analysis of NEDD9 and HER2 expression (Brown), Blue is DNA. NAT-Normal adjacent to tumor tissue. B. KM plotter analysis of NEDD9 expression in ER+ (left) and Triple Negative Breast Cancers. RFS-Relapse free Survival.

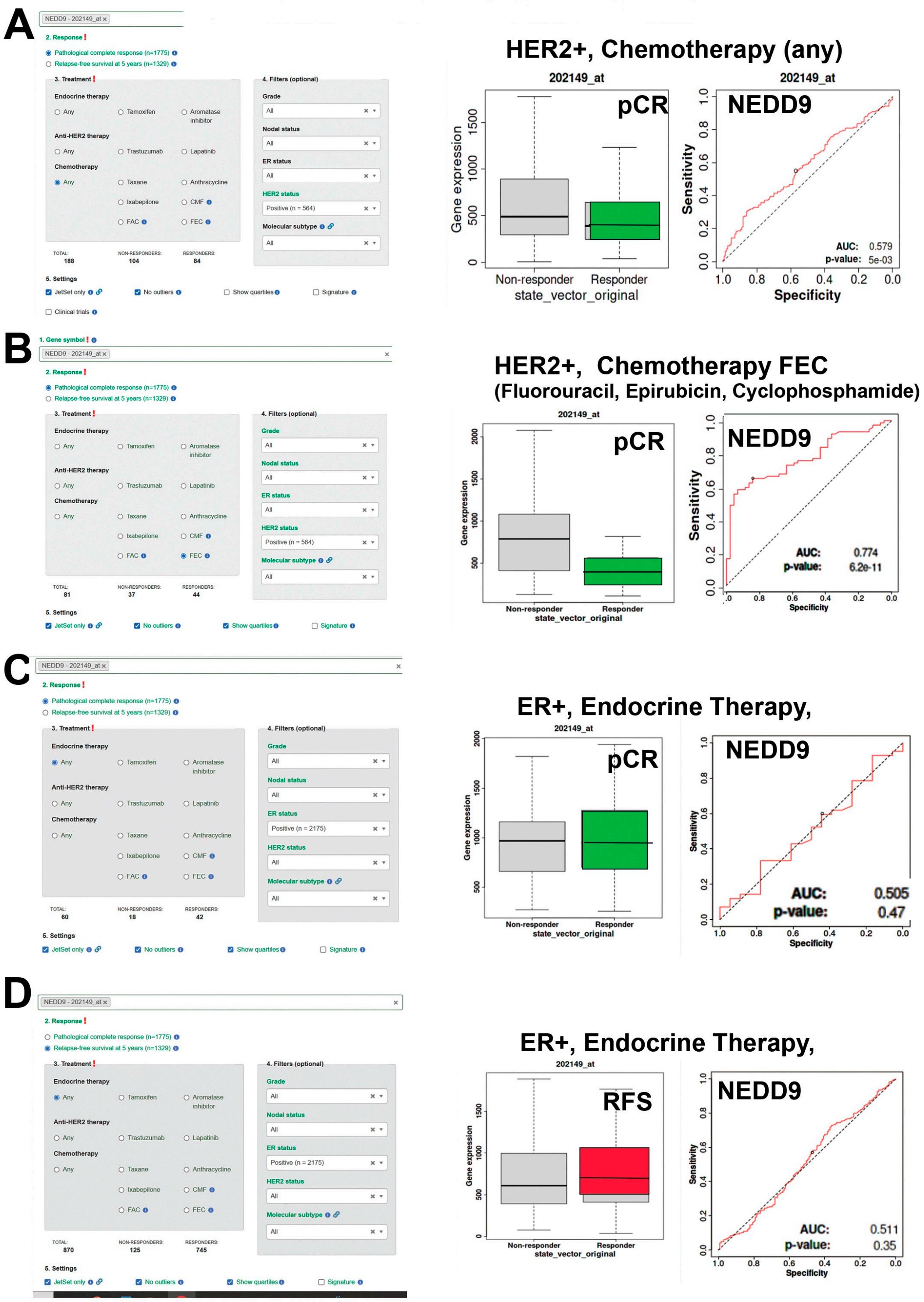

ROC plotter data analysis. The selection criteria outlined in the table (left) for each panel.

Figure S2: ROC plotter analysis of NEDD9 expression in ER+ and TNBC breast cancers.

Age	Sex	Organ	Pathology diagnosis	Grade	ER	PR	HER2	Type
44	F	Breast	Fibroadenoma	-	2+,30%	3+,70%	0	benign
27	F	Breast	Fibroadenoma	-	1+,5%	1+,3%	0	benign
23	F	Breast	Fibroadenoma	-	3+,80%	3+,30%	0	benign
19	F	Breast	Fibroadenoma	-	3+,95%	3+,90%	0	benign
23	F	Breast	Fibroadenoma	-	3+,95%	3+,95%	0	benign
25	F	Breast	Fibroadenoma	-	3+,95%	3+,95%	0	benign
23	F	Breast	Fibroadenoma	-			0	benign
42	F	Breast	Fibroadenoma	-			0	benign
48	F	Breast	Mild hyperplasia of breast duct	-	2+,40%	1+,3%	0	hyperplasia
41	F	Breast	Adenosis with hyperplasia of duct	-	+	+	0	hyperplasia
40	F	Breast	Atypical hyperplasia in duct	2	3+,95%	3+,95%	0	hyperplasia
76	F	Breast	Hyperplasia	-	++	+	0	hyperplasia
37	F	Breast	Adenosis with mild hyperplasia of duct	-	3+,90%	3+,60%	0	hyperplasia
45	F	Breast	Adenosis with mild hyperplasia of duct	-	3+,90%	3+,90%	0	hyperplasia
40	F	Breast	Cyclomastopathy	-	+	+	0	hyperplasia
43	F	Breast	Adenosis with hyperplasia of duct	-			0	hyperplasia
34	F	Breast	Mild hyperplasia of breast duct	-			0	hyperplasia
70	F	Breast	Hyperplasia	-			0	hyperplasia
32	F	Breast	Hyperplasia	-			0	hyperplasia
48	F	Breast	Hyperplasia	-			0	hyperplasia
45	F	Breast	Atypical hyperplasia of duct (grade II-III)	-			0	hyperplasia
22	F	Breast	Cyclomastopathy	-			0	hyperplasia
28	F	Breast	Cyclomastopathy	-			0	hyperplasia
35	F	Breast	Hyperplasia	-	+		0	hyperplasia
31	F	Breast	Cancer adjacent normal breast tissue (cyclomastopathy)	-	-	-	0	NAT
53	F	Breast	Cancer adjacent normal breast tissue	-	-		0	NAT
35	F	Breast	Cancer adjacent normal breast tissue	-	3+,80%	3+,60%	0	NAT
61	F	Breast	Cancer adjacent normal breast tissue	-			0	NAT
45	F	Breast	Cancer adjacent normal breast tissue	-			0	NAT
36	F	Breast	Cancer adjacent normal breast tissue	-			0	NAT
44	F	Breast	Cancer adjacent normal breast tissue	-			0	NAT
33	F	Breast	Cancer adjacent normal breast tissue	-			0	NAT

58	F	Breast	Cancer adjacent normal breast tissue	-			0	NAT
46	F	Breast	Cancer adjacent normal breast tissue	-	2+,30%	1+	0	NAT
15	F	Breast	Normal breast tissue	-			0	normal
21	F	Breast	Normal breast tissue	-			0	normal
21	F	Breast	Normal breast tissue	-			0	normal
35	F	Breast	Normal breast tissue	-			0	normal
19	F	Breast	Normal breast tissue	-			0	normal
27	F	Breast	Normal breast tissue	-			0	normal
44	F	Breast	Invasive ductal carcinoma	1	3+,95%	**2+,90%**	1+	malignant
50	F	Breast	Invasive ductal carcinoma	2	0	0	1+	malignant
48	F	Breast	Invasive ductal carcinoma	2	1+,60%	**2+,90%**	1+	malignant
62	F	Breast	Invasive ductal carcinoma	2	0	0	1+	malignant
68	F	Breast	Invasive ductal carcinoma	2	3+,95%	**3+,90%**	1+	malignant
44	F	Breast	Invasive ductal carcinoma	2	2+,90%	**2+,70%**	1+	malignant
54	F	Breast	Invasive ductal carcinoma	2	**3+,90%**	2+,10%	1+	malignant
55	F	Breast	Invasive ductal carcinoma	2	**3+,95%**	1+,5%	1+	malignant
43	F	Breast	Invasive ductal carcinoma	2	**3+,95%**	**3+,100%**	1+	malignant
62	F	Breast	Invasive ductal carcinoma	2	++	++	1+	malignant
45	F	Breast	Invasive ductal carcinoma	2	3+,95%	2+,50%	1+	malignant
55	F	Breast	Invasive ductal carcinoma	2	0	0	1+	malignant
69	F	Breast	Invasive ductal carcinoma	-	2+,50%	0	1+	malignant
70	F	Breast	Invasive ductal carcinoma	-	1+,40%	0	1+	malignant
71	F	Breast	Invasive ductal carcinoma	2	3+,100%	0	2+	malignant
38	F	Breast	Invasive ductal carcinoma	2	++	-	2+	malignant
41	F	Breast	Invasive ductal carcinoma	2	++	+	2+	malignant
32	F	Breast	Invasive ductal carcinoma	2	3+,95%	3+,90%	2+	malignant
63	F	Breast	Invasive ductal carcinoma	2	3+,95%	3+,70%	2+	malignant
59	F	Breast	Invasive ductal carcinoma	-	3+,80%	3+,80%	2+	malignant
65	F	Breast	Invasive ductal carcinoma	2	2+,80%	3+,80%	3+	malignant
54	F	Breast	Invasive ductal carcinoma	2	3+,95%	3+,30%	3+	malignant
50	F	Breast	Invasive ductal carcinoma	-	0	0	3+	malignant
35	F	Breast	Invasive ductal carcinoma	2	2+,70%	2+,40%	3+	malignant
29	F	Breast	Invasive ductal carcinoma	2	0	0	3+	malignant
28	F	Breast	Invasive ductal carcinoma	2	0	0	3+	malignant
55	F	Breast	Invasive ductal carcinoma	2	2+,40%	0	3+	malignant
52	F	Breast	Invasive ductal carcinoma	2	3+,100%	1+,3%	3+	malignant
38	F	Breast	Invasive ductal carcinoma	2	0	3+,5%	3+	malignant
49	F	Breast	Invasive ductal carcinoma	-	-	-	3+	malignant
46	F	Breast	Invasive ductal carcinoma	2	0	0	3+	malignant

29	F	Breast	Invasive ductal carcinoma	2	-	-	3+	malignant
51	F	Breast	Invasive ductal carcinoma	3	0	0	3+	malignant
62	F	Breast	Invasive ductal carcinoma	2	0	0	3+	malignant
53	F	Breast	Invasive ductal carcinoma	2	0	0	3+	malignant
52	F	Breast	Invasive ductal carcinoma	-	-	-	3+	malignant
40	F	Lymph node	Metastatic invasive ductal carcinoma	2	0	0	3+	metastasis
48	F	Lymph node	Metastatic invasive ductal carcinoma	-	1+,3%	3+,80%	3+	metastasis
56	F	Lymph node	Metastatic invasive ductal carcinoma	2	0	0	3+	metastasis
49	F	Lymph node	Metastatic invasive ductal carcinoma	2	0	0	3+	metastasis
66	F	Lymph node	Metastatic invasive ductal carcinoma	2	3+,100%	2+,60%	3+	metastasis
53	F	Lymph node	Metastatic invasive ductal carcinoma	2	0	0	3+	metastasis
52	F	Lymph node	Metastatic invasive ductal carcinoma	2	3+,100%	1+,3%	3+	metastasis
52	F	Lymph node	Metastatic invasive ductal carcinoma	2	2+,5%	0	3+	metastasis
28	F	Lymph node	Metastatic invasive ductal carcinoma	3	0	0	3+	metastasis
42	F	Lymph node	Metastatic invasive ductal carcinoma	2	0	0	3+	metastasis
80	F	Lymph node	Metastatic invasive ductal carcinoma	2	-	0	3+	metastasis
59	F	Lymph node	Metastatic invasive ductal carcinoma	2	3+,40%	0	3+	metastasis
60	F	Lymph node	Metastatic invasive ductal carcinoma	2	0	0	3+	metastasis
28	F	Lymph node	Metastatic invasive ductal carcinoma	2	0	0	3+	metastasis

Table S1. Tissue pathology and diagnosis used in Figure 1.

Figure S3. The original western blots of Figure 6.

Anti-HER2

kDa

200

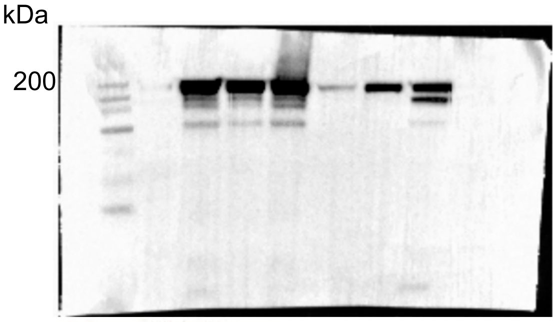

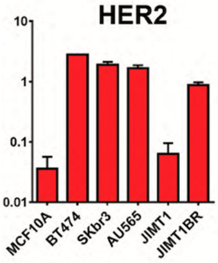

Anti-NEDD9

kDa

115

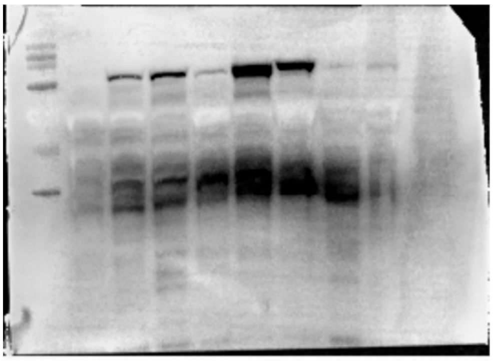

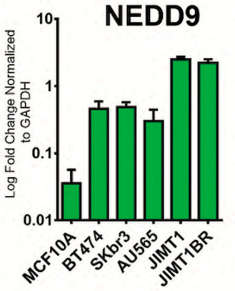

Anti- GAPDH

kDa

40

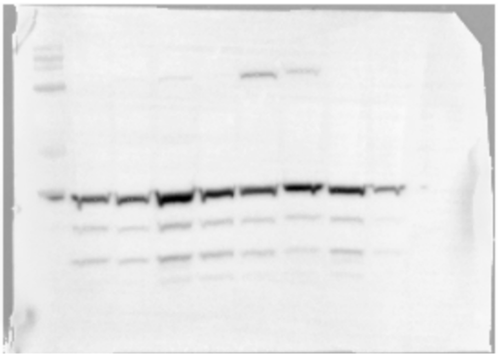

AU565 and JIMT1

Anti-NEDD9

kDa

115

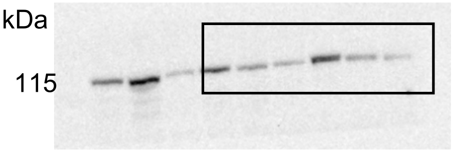

Anti-GAPDH

kDa

40

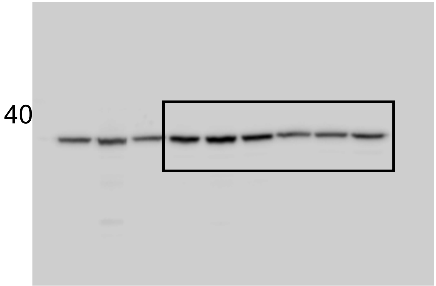

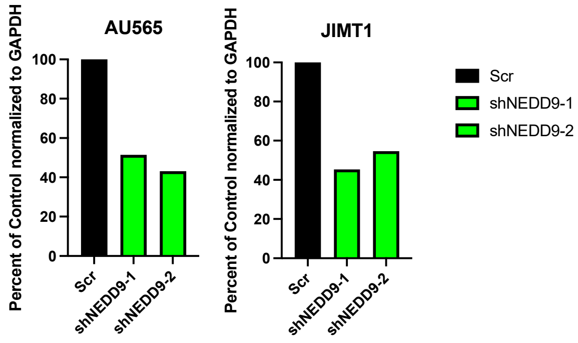

BT474

Anti-NEDD9

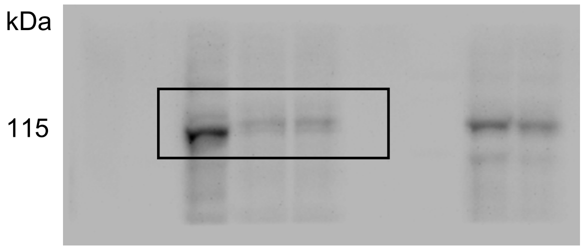

Anti-GAPDH

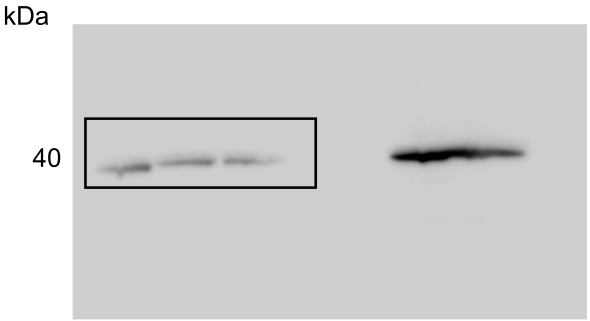

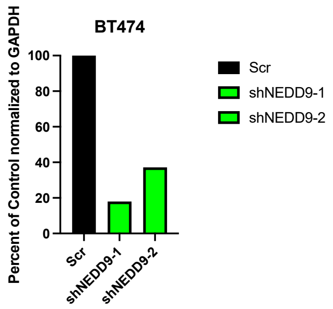

Figure S4. The original western blots of Figure 7.

Anti-NEDD9

kDa

115

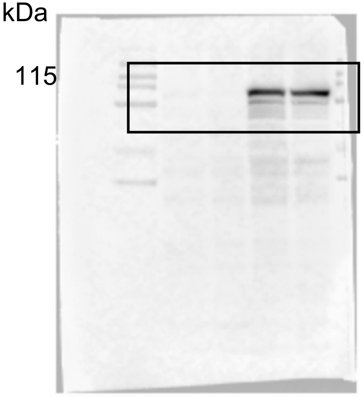

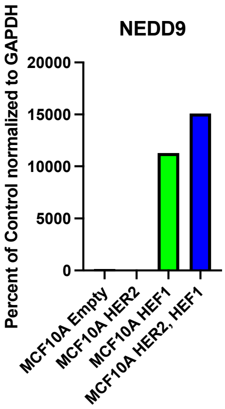

Anti-HER2

kDa

200

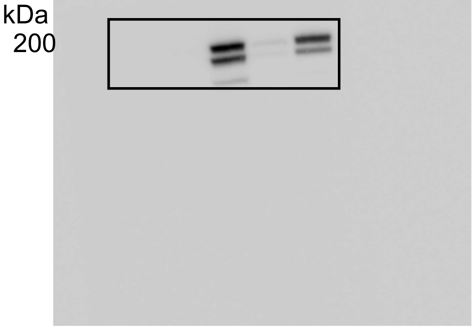

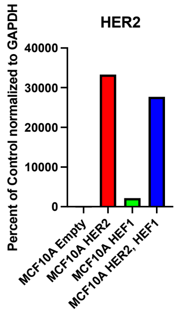

Anti-pERK

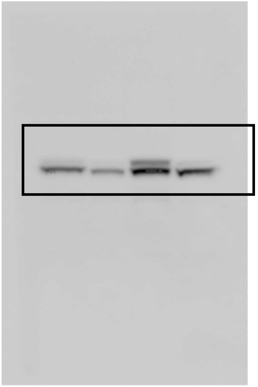

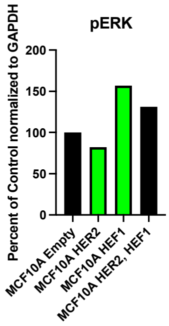

Anti-tERK

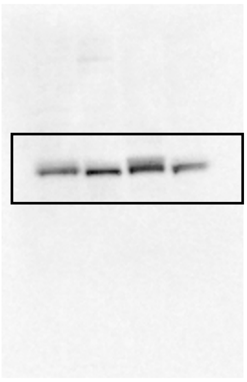

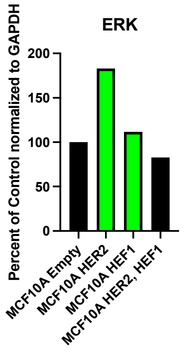

Anti-GAPDH

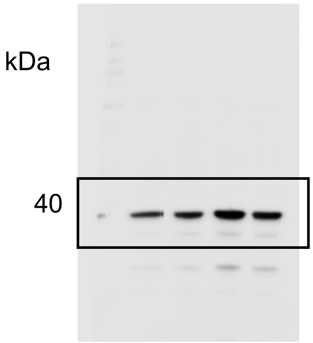

Anti-pAURKA

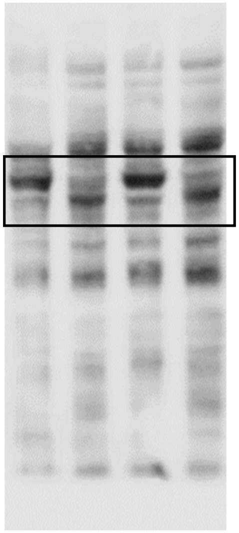

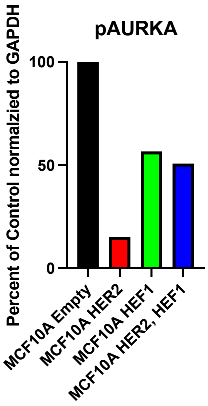

AURKA

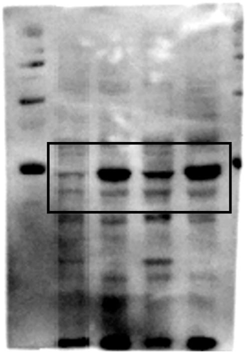

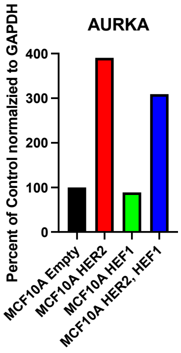

GAPDH

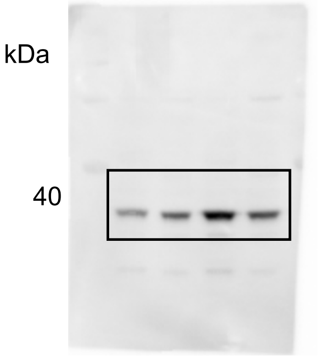

pFAK

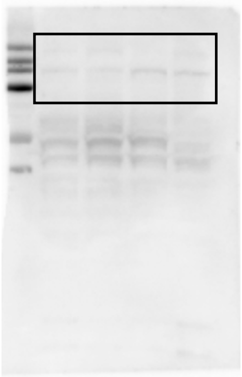

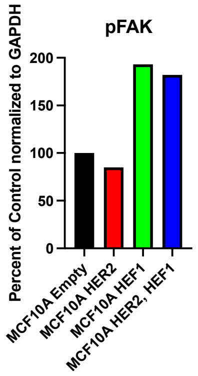
pFAK
Percent of Control normalized to GAPDH
200
150
100
50
0
MCF10A Empty
MCF10A HER2
MCF10A HEF1
MCF10A HER2, HEF1

FAK

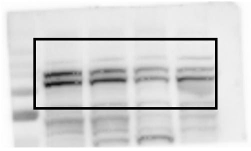

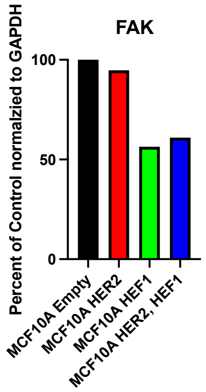
FAK
Percent of Control normalzied to GAPDH
100
50
0
MCF10A Empty
MCF10A HER2
MCF10A HEF1
MCF10A HER2, HEF1

pSRC

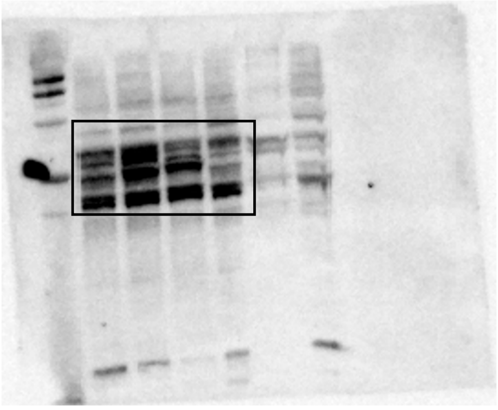

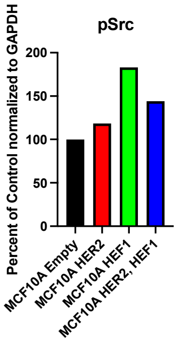
pSrc
Percent of Control normalized to GAPDH
200
150
100
50
0
MCF10A Empty
MCF10A HER2
MCF10A HEF1
MCF10A HER2, HEF1

SRC

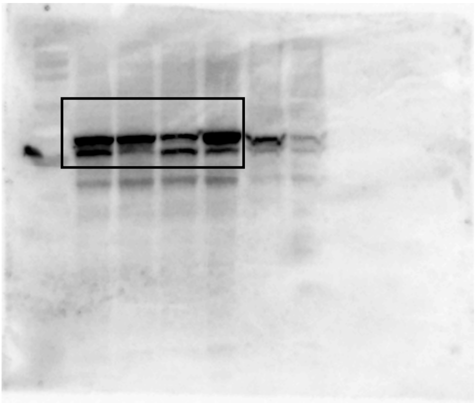

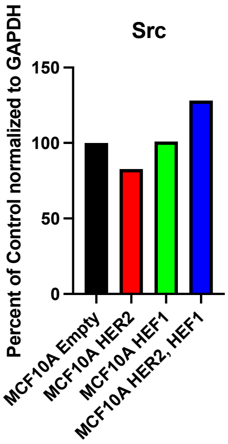
Src
Percent of Control normalized to GAPDH
150
100
50
0
MCF10A Empty
MCF10A HER2
MCF10A HEF1
MCF10A HER2, HEF1

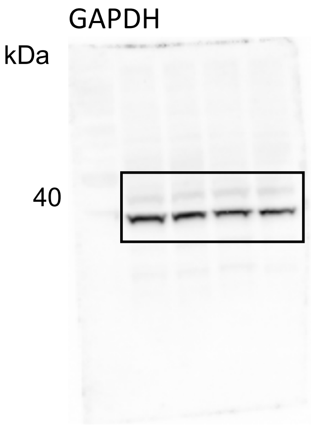
GAPDH
kDa
40
